# A Report of a Large Axillary Cystic Hygroma (a.k.a Lymphangioma) in a Newborn from a Tertiary Hospital in Northern Tanzania

**DOI:** 10.1155/2020/5624019

**Published:** 2020-11-17

**Authors:** Jay Lodhia, Rune Philemon, Patrick Amsi, Kondo Chilonga, David Msuya

**Affiliations:** ^1^Department of General Surgery, Kilimanjaro Christian Medical Center, P O Box 3010, Moshi, Tanzania; ^2^Kilimanjaro Christian Medical University College, P O Box 2240, Moshi, Tanzania; ^3^Department of Pathology, Kilimanjaro Christian Medical Center, P O Box 3010, Moshi, Tanzania; ^4^Department of Pediatrics, Kilimanjaro Christian Medical Center, P O Box 3010, Moshi, Tanzania

## Abstract

**Introduction:**

Cystic hygroma is a rare condition of the lymphatic system that occurs mainly in children. They are found around the neck, axilla, inguinal, or thoracic regions. *Case Presentation*. A newborn female baby with a right-sided axillary mass since birth was admitted to our center. She was otherwise a healthy baby with noncontributory prenatal history. The mass was 12 cm in diameter and cystic. Wide excision of the mass was done, and histology confirmed cystic hygroma. Postoperatively, the baby did well clinically and was discharged.

**Conclusion:**

Due to its rare incidence, reports and literature on management of cystic hygroma are few. A multidisciplinary approach is vital to yield the best prognosis for this rare condition.

## 1. Introduction

Cystic hygroma, also known as lymphangioma, is caused by lymphatic malformations which present as fluid-filled lesions [1]. Cystic hygromas are mostly located on the neck, accounting for 75%, followed by the axilla at 20%, and about 1% in the mediastinum [1]. This condition can also be accompanied by chromosome aneuploidies, hydrops fetalis, and intrauterine fetal death [1]. Cystic hygroma can be diagnosed intrauterine by obstetric ultrasonography and managed pharmacologically. It can regress spontaneously with the advancement of pregnancy due to the maturation of normal lymphatics [1].

## 2. Case Presentation

**Figure 1:**
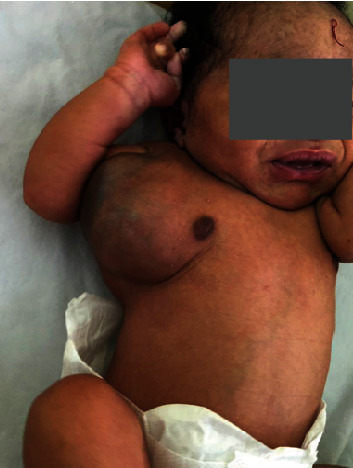
Photograph showing left axillary cystic hygroma.

A one-day-old female baby who was born with a mass in her right axilla was referred to our center. The baby was delivered by spontaneous vaginal delivery with APGAR scores of 9 and 10 in the first and fifth minutes, respectively, and birth weight of 3 kilograms. The mother reported normal pregnancy with no complications with normal obstetric ultrasound at prenatal visits. Upon initial examination, the baby looked otherwise healthy, breastfed actively, and was afebrile. There was an obvious swelling in the right axillary with hyperpigmented skin over it and visible superficial veins (Figure 1). The mass was 12 cm in diameter, soft, cystic, nontender, not warm, and not pulsating. No bruits were heard on auscultation over the swelling.

**Figure 2:**
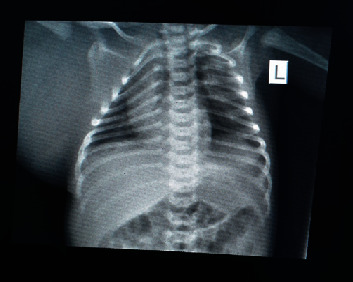
Plain chest X-ray showing homogenous soft tissue lesion left axillary region.

A plain chest X-ray was done that concluded normal chest cage with a normal cardiac shadow with a homogenous dense right axillary region with no bony abnormalities (Figure 2). Ultrasonography of the mass was done and concluded that the mass had cystic and solid components, was vascularized, and had no clear origin. It was located adjacent to the parietal pleura, but no pleural fluid collection was noted. The axillary vessels were sonographically normal.

**Figure 3:**
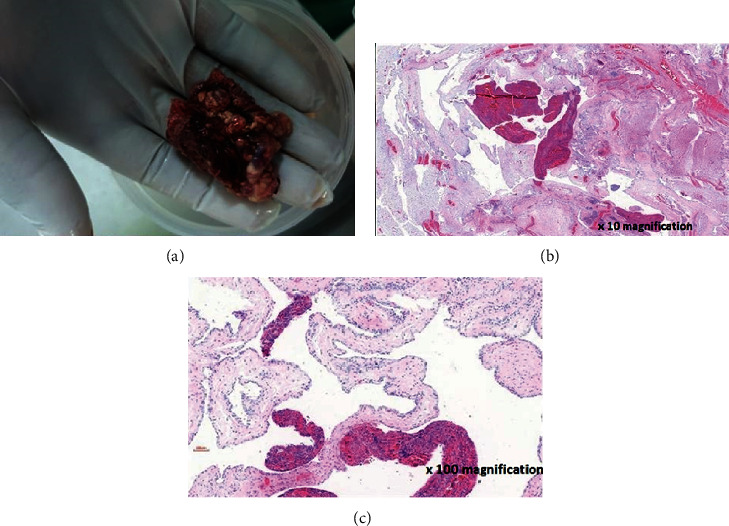
(a) Excised lesion; (b, c) H & E stained sections of the neck mass showing large, irregularly formed vascular spaces which are lined by flattened, bland epithelial cells embedded within a fibrous stroma.

The baby was prepared for surgery; blood parameters were within range, and vitals were normal. Wide local excision was done with primary closure. The baby recovered well and was discharged after ten days with no complications. The histology results confirmed features of cystic hygroma (Figure 3). The baby was reviewed three weeks postsurgery at the surgical outpatient clinic and was clinically stable, had no arm oedema, and surgical incision scar had healed.

## 3. Discussion

Cystic hygroma, also known as lymphangioma, is an uncommon benign congenital anomaly whereby the lymphatic vessels are maldeveloped [2]. This occurs mostly during childhood and accounts for 6% of all pediatric soft tissue tumors [3]. The incidence is said to be 1/6000 at birth and 1/750 in spontaneous abortion [1]. Most affected areas are cervical and axilla. Rarely, it affects the inguinal, retroperitoneal, and thoracic regions [3]. The commonest site is the posterior triangle of the neck due to the embryological development of the lymphatics [4]. The masses or lesions exert as solitary fluctuant, compressible, movable, and painless in characteristics as this was the case in our newborn baby, though sizes can vary [3]. Sometimes they can bleed and increase in size, and thus compress local organs or develop infection [4].

They are divided into two histological types based on the size of the abnormal lymph vessels and depth. Superficial ones are called lymphangioma circumscriptum, and cavernous lymphangioma or cystic hygroma are the deeper seated [4]. They are mostly found at birth (50% in affected newborns). They are usually multiloculated and tend to grow if not managed timely [4]. They can also coexist with other syndromes such as Noonan syndrome, Turner syndrome, fetal alcohol syndrome, or chromosomal aneuploidy [4, 5]. With regard to the index case, there were no other obvious malformations noted phenotypically, and genetic evaluation was not done due to the financial circumstances and unavailability of the services in our setting.

Examination and a wide range of investigations should be performed to rule out other differential diagnoses such as lipoma, teratoma, hemangioma, or even high thoracic meningomyelocele [4, 6]. Ultrasonography and Doppler ultrasound can be ideal as it is cost-effective, nonradiation, and noninvasive and should be sought particularly in young children with similar conditions [6, 7]. Computed tomography or magnetic resonance imagining can be done at the discretion of the clinicians to come to a definitive diagnosis and aid in the management [8].

Surgery remains the most preferred management of cystic hygroma [9]. Clinicians can opt for watchful waiting for up to two years as there are some reports of spontaneous regression [4]. Other modalities include aspiration or injection of sclerosing agents like OK-432, fibrin adhesives, bleomycin, hypertonic saline, or alcohol [1, 2, 4, 5, 9]. These noninvasive therapies can be sought if there are other contraindications for anaesthesia, high-risk surgery, or to down-stage the size of the lesion. As noted in our case, surgery was performed successfully with good recovery and diagnosis was also confirmed by histology analysis of the resected mass.

Other therapies mentioned by Ersoy et al. are the use of propranolol and sirolimus, which are VEGF inhibitors and promote apoptosis of lymphangioma cells. The authors mention the successful use of this drug in making the lesion significantly smaller in size [10].

## 4. Conclusion

Management of cystic hygromas remains a challenge, especially those that are large and located in crucial areas, which then might exert complications for surgery and anaesthesia. Nevertheless, medical and surgical therapy can be sought depending on the clinicians' decision and experience with good prognosis.
